# Interventional management of unresectable pancreatic cancer using transarterial chemoembolization and microwave ablation: a single-center evaluation over 12 years

**DOI:** 10.1007/s00432-026-06463-3

**Published:** 2026-03-31

**Authors:** Thomas J. Vogl, Ruxandra Cojocaru, Daniel M. Dahm, Hamzah Adwan

**Affiliations:** Clinic for Radiology and Nuclear Medicine, University Hospital Frankfurt, Goethe University, Theodor-Stern-Kai 7, 60590 Frankfurt Am Main, Germany

**Keywords:** Unresectable pancreatic cancer, Microwave ablation, Transarterial chemoembolization, Locoregional therapy, Interventional oncology, Overall survival

## Abstract

**Purpose:**

The aim of this study is to evaluate the safety and clinical outcomes of combined transarterial chemoembolization (TACE) and microwave ablation (MWA) as well as TACE monotherapy in patients with unresectable pancreatic cancer.

**Materials and methods:**

Between 2010 and 2023, 150 patients with unresectable locally advanced pancreatic cancer were included in this retrospective single-center study and treated with either combined TACE and MWA (Group A, n = 67) or TACE alone (Group B, n = 83). In Group A, 23/67 (34.3%) patients presented with metastatic disease and in Group B 45/83 (54.2%) patients had metastatic disease. A total of 222 TACE procedures and 71 MWA procedures were performed in Group A, while 250 TACE procedures were performed in Group B. Follow-up was performed using contrast-enhanced cross-sectional imaging and clinical evaluation. Tumor response and overall survival were analyzed.

**Results:**

The mean tumor diameter was 3.4 ± 0.9 cm in Group A and 4.9 ± 1.3 cm in Group B. No major complications occurred in either treatment group. Minor hemorrhage following MWA was observed in 4/71 ablations (5.6%) without further clinical consequence. In Group A, follow-up imaging was available for 47/67 patients, with local tumor progression observed in 5/47 patients (10.6%). The 1-year overall survival rate was 69.4%, with a median overall survival time of 14.6 months (95% CI 12.6–16.7 months). In Group B, follow-up imaging was available for all 83 patients, with local tumor progression observed in 8/83 patients (9.6%). The 1-year overall survival rate was 43.6%, with a median overall survival time of 9.0 months (95% CI 3.3–14.7 months).

**Conclusion:**

TACE and MWA were safe locoregional treatments for patients with unresectable pancreatic cancer. Both treatment protocols, including the combination of TACE with MWA and TACE as monotherapy, were effective and showed promising results.

## Introduction

Pancreatic cancer is an aggressive disease associated with a disproportionally high mortality. According to recent statistics, an estimated 66.440 new cases of pancreatic cancer and 51.750 related deaths were expected in the United States in 2024, when considering both sexes combined (Siegel et al. 2024). This close alignment between incidence and mortality reflects the particularly poor prognosis of the disease. Five-year survival rate remains low overall at approximately 9%, with prognosis strongly depending on the stage at diagnosis (Rawla et al. 2019). The only potentially curative treatment for pancreatic cancer is surgical resection (Kamisawa et al. 2016). At present, the available treatment for unresectable pancreatic cancer mainly consists of chemotherapy, a combination therapy of 5-fluorouracil, oxaliplatin, irinotecan and leucovorin (FOLFIRINOX) or a monotherapy with Gemcitabine (Tempero et al. 2021).

In recent years, the role of interventional radiology in oncologic treatment has gained significance, leading to studies evaluating ablative methods for pancreatic cancer treatment.

However, most studies have focused on methods such as radiofrequency ablation (RFA), irreversible electroporation (IRE), or a combination of RFA and transarterial chemoembolization (TACE). The available literature on microwave ablation (MWA) is limited. An overview published in 2020 included only 3 papers evaluating the use of MWA each involving a small number of patients (maximum of 26), whilst 18 papers on the use of RFA and 10 papers on the use of IRE in pancreatic cancer could be included (Granata et al. 2020). Another review published identified only 4 studies evaluating MWA, all of which reported limited follow-up and survival data (Punzi et al. 2023). TACE has been used in multiple studies on liver metastasis from pancreatic cancer (Timmer et al. 2021a) yet the data available on TACE as monotherapy on pancreatic cancer is also poor (Das et al. 2019).

TACE is an interventional radiology procedure used for tumors. It involves selective catheterization of the tumor-feeding artery, followed by intra-arterial delivery of chemotherapeutic agents and embolic materials. This achieves high local drug concentration while inducing ischemia, effectively targeting the tumor with minimal systemic toxicity (Timmer et al. 2021b).

MWA is a minimally invasive thermal ablation technique, which induces coagulative necrosis in tumor tissue. Under image guidance, a microwave antenna is placed into or adjacent to the lesion. The emitted energy generates localized heat, effectively destroying the target tissue while preserving surrounding structures (Simon et al. 2005).

In this paper, our focus is on a combination therapy comprising TACE and MWA, as well as TACE as monotherapy, in treating patients with unresectable locally advanced pancreatic cancer. This study aimed to further characterize the safety profile and clinical outcomes of combined TACE and MWA compared with TACE monotherapy in patients with unresectable pancreatic cancer.

## Materials and methods

This retrospective single-center study was performed at our university hospital. The study was conducted according to the guidelines of the Declaration of Helsinki and approved by the Ethics Committee of the Faculty of Medicine at Goethe University in Frankfurt am Main. The requirement for informed consent was waived due to the retrospective nature of the study. This study includes interventional treatments of patients with unresectable locally advanced adenocarcinoma of the pancreas at our hospital between 2010 and 2023 which have been recorded in an institute’s internal database. After performing a pre-treatment contrast-enhenced MRI of the abdomen and a Chest-CT, the main reason for unresectability was locally advanced disease with arterial and/or venous involvement. In addition to a locally advanced disease, some patients also presented with distant metastasis. The patients had received first-line systemic therapy prior to interventional treatments.

To ensure consistency and data integrity, patients were included in the study if they met the following criteria: (1) Availability of essential clinical documentation, specifically: date of first treatment; date of last treatment; date of last contact or date of death; (2) Contrast- enhanced cross-sectional imaging was performed to enable assessment of tumor stage; (3) histological diagnosis was performed under endoscopic ultrasound-guidance or percutaneously under CT-guidance of adenocarcinoma of the pancreas as the only known primary malignancy; (4) the pancreatic tumor was ruled unresectable by a multidisciplinary tumor board based on imaging findings and clinical assessment.

Patients were excluded from the analysis if they met any of the following criteria: (1) evidence of impaired coagulation, defined as an international normalized ratio (INR) greater than 1.5 or a platelet count below 50,000/µL; (2) presence of any other known primary tumor; or (3) additional treatment with RFA.

Patients were divided into two groups: Group A, treated with combined TACE and MWA, and Group B, treated with TACE monotherapy. The decision of whether patients were treated by TACE and MWA or TACE alone was taken in a multidisciplinary tumor board. Generally, patients with lower tumor burden were treated by the combination treatment protocol and patients with larger tumors were usually treated by TACE alone. Accordingly, baseline differences between groups were expected and no formal comparative efficacy conclusion between treatment protocols was intended. The aim of both treatment protocols was to mainly achieve local tumor control.

### Interventional procedures

TACE and MWA were performed as locoregional interventional treatment modalities in patients with unresectable pancreatic cancer.

For TACE, local anesthesia was applied to the inguinal region following sterilization and draping, after which the common femoral artery was punctured. After that, angiograms of the abdominal aorta as well as celiac trunk and mesenteric superior artery were obtained. Depending on tumor localization and arterial supply, catheterization of the splenic artery, gastroduodenal artery or inferior pancreaticoduodenal artery was subsequently performed. The chemotherapeutic regimen administered during TACE consisted of either (1) mitomycin, irinotecan, and cisplatin or (2) gemcitabine, irinotecan, and cisplatin. Lipiodol and Embocept were used for embolization of the tumor-feeding artery (Vogl et al. 2023).

MWA was performed percutaneously under CT guidance in all cases. Pre-interventional imaging was reviewed on the day of the procedure to assess lesion size and anatomical location, and immediate pre-ablation imaging was acquired using a 64-slice multidetector CT scanner. Intravenous sedative and analgesic medication were administered before and during the procedure, with continuous monitoring of blood pressure, ECG, and pulse oximetry under aseptic conditions. Following a small skin incision, the microwave antenna was inserted under CT guidance. During ablation, intermittent unenhanced CT images were obtained to monitor the procedure and detect complications. After completion of tumor ablation, antenna tract cauterization was performed using thermal coagulation to prevent tumor cell seeding. Patients remained on bed rest for the first 8 h after the procedure under close clinical observation to monitor vital signs and pain levels.

Tumor response was assessed using contrast-enhanced cross-sectional imaging after a TACE or MWA session.

Major complications were defined in accordance with the Society of Interventional Radiology (SIR) classification system as events associated with substantial morbidity or disability, the need for an increased level of care, or prolonged hospitalization, while all remaining events were classified as minor complications (Sacks et al. 2003).

### Statistics

Raw data were extracted using Microsoft Excel. Excel tables were used as input files for statistical analysis using SPSS. Normally distributed parameters were tested with a two-tailed *t-test*. Non-normally distributed parameters were analyzed with the Mann-Whitney U test. Categorical data were expressed as frequencies and percentages. Survival rates were calculated using the Kaplan-Meier survival estimator (Kaplan and Meier 1958; Bewick et al. 2004). For both groups, complication rate, local tumor progression rate, distant tumor progression rate, and overall survival were calculated.

The first interventional therapy, TACE, is the starting point for overall survival calculations.

The end date for overall survival was defined as the date of death from any cause. Patients who were still alive at the time of last follow-up were censored at the date of last contact (Puijk et al. 2021; Delgado and Guddati 2021).

## Results

### Patient population and baseline characteristics

The study included a total of 150 patients with unresectable pancreatic cancer. Sixty-seven patients underwent combined treatment with TACE and MWA (Group A), while 83 patients were treated with TACE monotherapy (Group B). Baseline demographic and clinical characteristics of both groups are summarized in Table 1.

**Table 1:** Baseline characteristics of the study population

Characteristics	Group A: TACE and MWA (*n* = 67)	Group B: TACE only (*n* = 83)
Age, years (mean)	59.2	64.0
*Sex, n (%)* Male Female	40 (59.7) 27 (40.3)	40 (48.2) 43 (51.8)
Metastatic disease, n (%)	23 (34.3)	45 (54.2)
Initial tumor diameter, cm (mean$\pm$SD)	3.4 ± 0.9	4.9 ± 1.3

TACE , Transarterial chemoembolization; MWA , Microwave ablation

### Treatment characteristics and safety

Treatment characteristics and procedure-related complications are summarized in Table 2. In Group A, a total of 222 TACE procedures and 71 MWA procedures were performed, with a mean of 3.3 TACEs per patient prior to ablation. Four patients (6.0%) underwent more than one MWA session. Initial complete ablation was achieved in 63/67 lesions (94.0%).

**Table 2:** Treatment characteristics and procedure-related complications

Parameter	Group A: TACE and MWA (*n* = 67)	Group B: TACE only (*n* = 83)
Total TACE procedures, n	222	250
TACEs per patient, mean	3.3	3.0
Total MWA procedures, n	71	–
Patients with > 1 MWA, n (%)	4 (6.0)	–
Initial complete ablation rate, n (%)	63/67 (94.0)	–
Major complications, n (%)	0 (0)	0 (0)
Minor complications, n (%)	4 (5.6)	0 (0)

TACE , Transarterial chemoembolization; MWA ,  Microwave ablation

No major complications occurred in either group. Minor hemorrhage following MWA was observed in 4/71 ablations (5.6%), all without further clinical consequence. No complications were recorded during TACE procedures in either group.

### Tumor progression

Follow-up with contrast-enhanced cross-sectional imaging was available in 47/67 patients in Group A and in all patients in Group B. Tumor evaluation was not possible for the remaining patients due to unavailable radiological data. Local tumor progression occurred in 5/47 patients (10.6%) in Group A and in 8/83 patients (9.6%) in Group B. Distant tumor progression was observed in 29/47 patients (61.7%) in Group A and in 28/83 patients (33.7%) in Group B.

### Overall survival

**Fig. 1:**
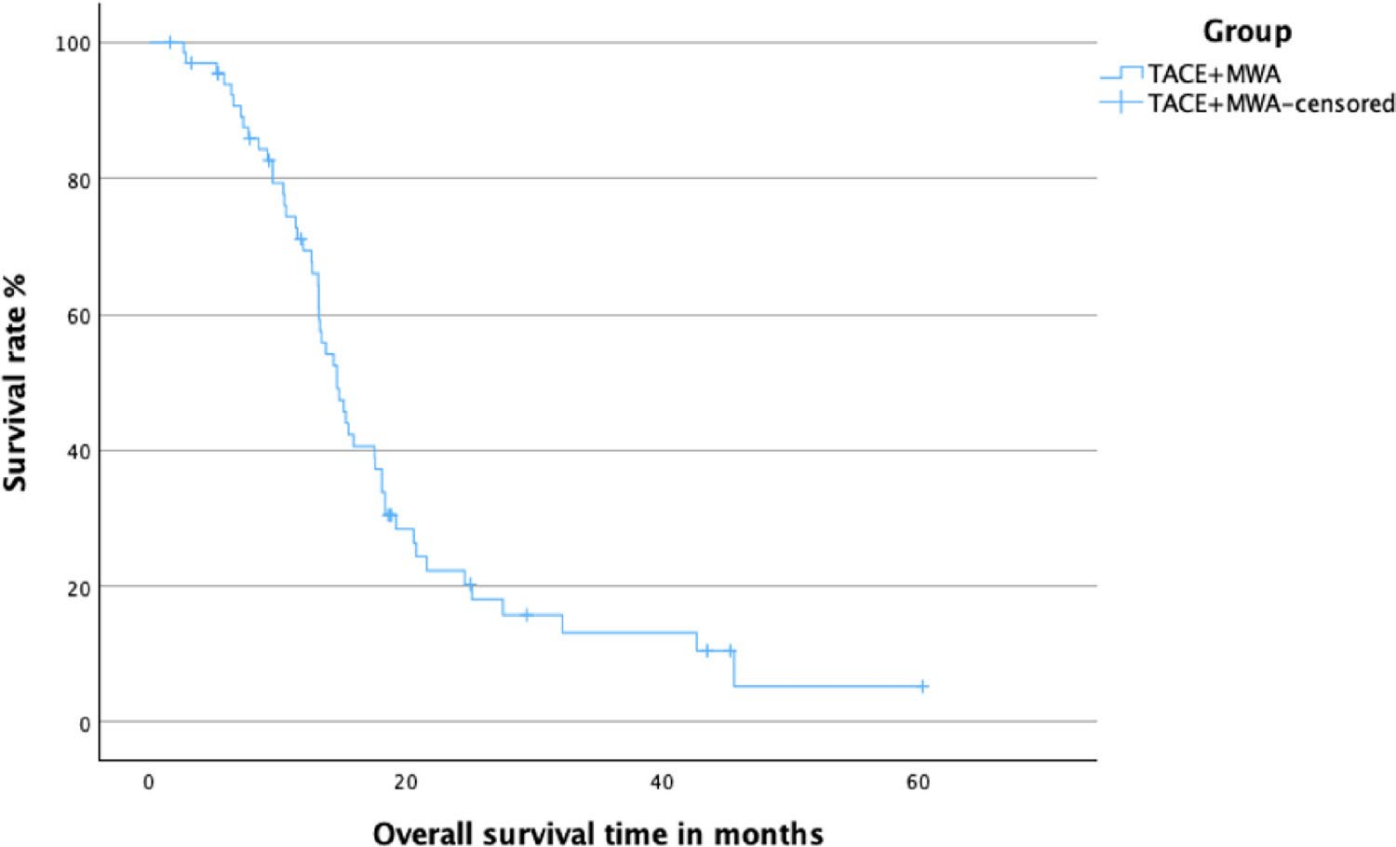
Kaplan-Meier curve of overall survival for Group A (combination therapy of TACE and MWA)

In Group A, the 1-year overall survival rate was 69.4%. The median overall survival time was 14.6 months (95% CI 12.6–16.7). The mean overall survival time was 19.3 months (95% CI 15.5–23.1). The corresponding Kaplan–Meier survival curve is shown in Fig. 1

**Fig. 2:**
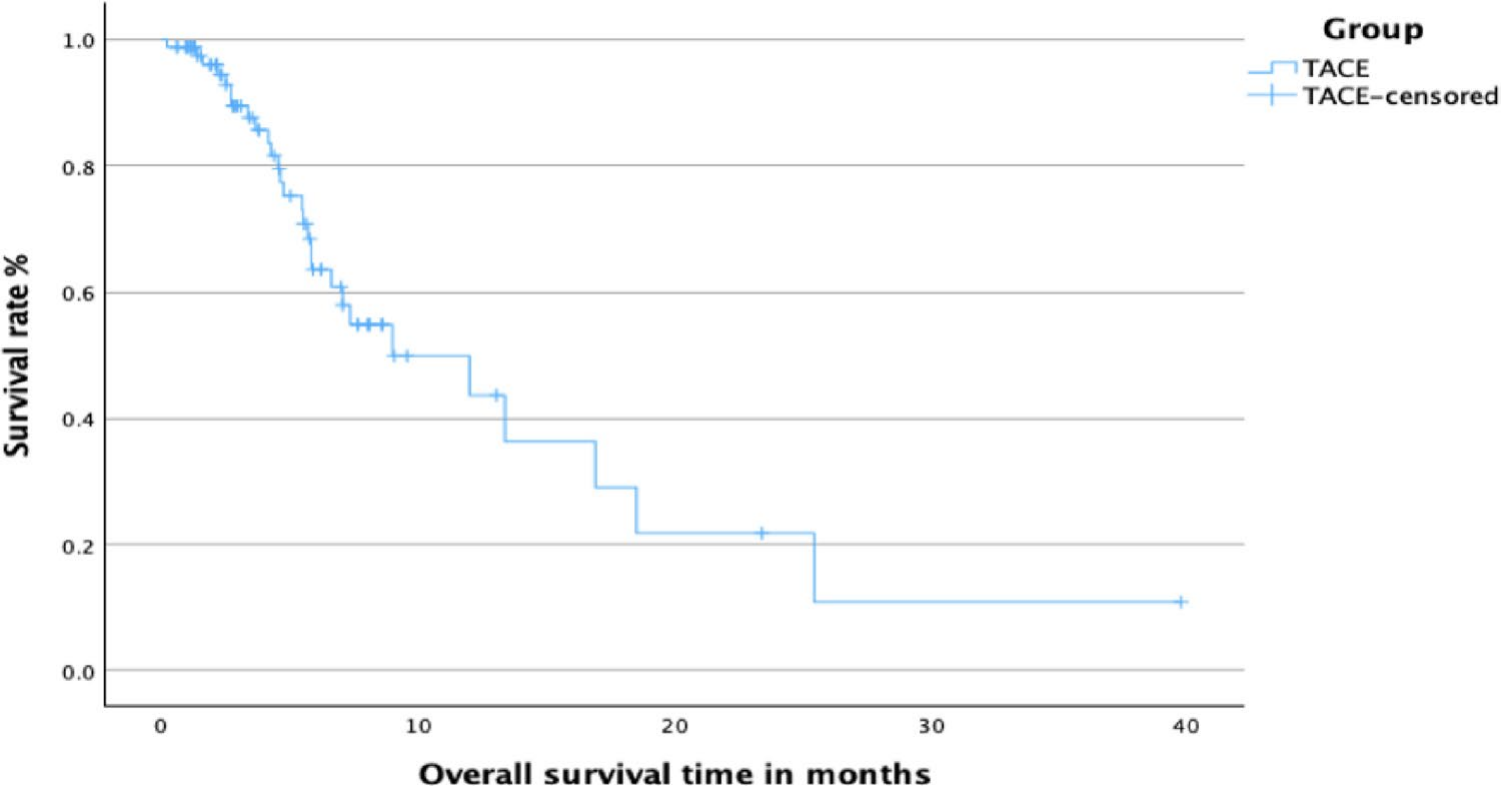
Kaplan-Meier curve of overall survival for Group B (Monotherapy with TACE)

 In Group B, the 1-year overall survival rate was 43.6%. The median overall survival time was 9.0 months (95% CI 3.3–14.7). The mean overall survival time was 13.9 months (95% CI 9.1–18.7). The corresponding Kaplan–Meier survival curve is presented in Fig. 2.

## Discussion

### Procedure-related safety

In this retrospective single-center cohort, TACE and MWA were associated with a favorable safety profile when applied in appropriately selected patients with unresectable pancreatic cancer. No major complications occurred following either procedure. Minor complications were observed in 4 of 71 MWA procedures (5.6%) and consisted exclusively of local hemorrhage, all of which resolved without the need for additional intervention. These findings are consistent with previously published reports on MWA in pancreatic cancer. Lygidakis et al. (2007) and Ierardi et al. (2018) reported no major complications following MWA, whereas Carrafiello et al. (2013) described one major complication of arterial pseudoaneurysm. In addition, Lygidakis et al. (2007) reported minor complications in 6 of 15 patients, including mild pancreatitis, asymptomatic hyperamylasemia, pancreatic ascites, and minor bleeding. When compared with (IRE), the complication rate observed in the present study was lower. Martin et al. (2015) reported a complication rate of 37% in a cohort of 200 patients treated with IRE for locally advanced pancreatic cancer, with a median complication grade of 2, while a review by Uysal et al. (2021) described complication rates ranging from 10% to 37%. Compared with RFA, the complication profile observed in the present study appears similar. D’Onofrio et al. (2017) reported no complications in 18 patients with locally advanced pancreatic cancer treated with RFA, whereas Zou et al. (2010) observed complications in 3 of 32 patients (9.4%), all of which resolved with conservative management. 

Data on transarterial local chemotherapy in pancreatic cancer remain limited. Vogl et al. (2007) reported no clinically relevant complications following transarterial chemoperfusion in 40 patients with locally recurrent pancreatic cancer. Das et al. (2019) described grade 3 complications in 28 of 143 patients (19.6%) treated with TACE, with no grade 4 events. Notably, tumors treated in that study were substantially larger than those included in the present cohort (mean diameter 9.9 cm vs. 4.9 cm), which may partly explain the higher complication rate.

### Oncological outcomes

To date, the number of studies investigating MWA in pancreatic cancer remains limited, typically comprising small patient cohorts with relatively short follow-up periods, and none have specifically evaluated the efficacy of combined TACE and MWA (Granata et al. 2020; Punzi et al. 2023). Ierardi et al. (2018) reported outcomes in five patients treated with MWA, observing no complete or partial responses at 12 months, with three patients dying during follow-up due to disease progression. In the present study, patients treated with combined TACE and MWA demonstrated a median overall survival time of 14.6 months (95% CI 12.6–16.7), with a 1-year overall survival rate of 69.4%. These outcomes were achieved in a cohort including unresectable locally advanced pancreatic cancers with 23 patients additionally having distant metastases. By comparison, Carrafiello et al. (2013) reported a 1-year survival rate of 80% following MWA in a highly selected population of patients with locally advanced disease only. Differences in patient selection and disease stage likely account for this discrepancy. Both external studies reported improvements in quality of life following treatment; however, quality-of-life parameters were not assessed in the present study. 

Evidence regarding TACE monotherapy in pancreatic cancer is similarly scarce. Das et al. (2019) reported slightly higher survival rate in a cohort of 59 patients with unresectable pancreatic cancer treated by TACE as monotherapy, with 1-year survival rate of 47.46%. In the present study, patients treated with TACE alone achieved 1-year overall survival rate of 43.6%. Differences in treatment protocols and patient populations may have contributed to these variations. 

When compared with RFA, survival outcomes in the combined TACE and MWA group appear comparable. D’Onofrio et al. (2017) reported a mean survival of 185 days following RFA in 18 patients, whereas a mean survival time of 19.3 months was observed in the present combination therapy cohort. Zou et al. (2010) reported 1-year survival rate of 65.6% following RFA in patients with locally advanced pancreatic cancer, which are similar to the corresponding rate observed in the present study. 

In comparison with IRE, median overall survival in the present study was lower. Martin et al. (2015) reported a median survival of 24.9 months in 200 patients treated with IRE for locally advanced pancreatic cancer, whereas the median survival time in the combined TACE and MWA group was 14.6 months. This difference may be attributable, at least in part, to the inclusion of patients with metastatic disease in the present cohort. The combination therapy group demonstrated survival outcomes exceeding those reported for systemic gemcitabine monotherapy. Das et al. (2019) showed 1-year survival rate of 23.57% in patients receiving gemcitabine alone. In contrast, higher survival rates were observed in both groups of the present study. However, we should interpret these findings cautiously, as we did not perform a direct comparison with systemic therapy.

When compared with reported outcomes for FOLFIRINOX, patients treated with combined TACE and MWA demonstrated a comparable median overall survival time of 14.6 months versus 11.1 months reported by Conroy et al. (2011), while patients treated with TACE alone showed a shorter median survival time of 9.0 months. Differences in patient selection and disease stage limit direct comparison.

### Limitations

This study has several limitations. Its retrospective design introduces inherent selection bias, and treatment allocation was not randomized but based on multidisciplinary tumor board decisions and clinical judgment. Accordingly, baseline differences between patient groups were present, and the study was not designed to allow a formal comparative evaluation of the two treatment approaches. We also did not have a control group that was treated by other treatment for comparison. The single-center setting and long inclusion period may have introduced additional variability related to evolving treatment strategies, imaging techniques, and supportive oncologic care. Follow-up imaging was not available for all patients. Furthermore, quality-of-life parameters were not systematically collected and therefore could not be analyzed.

## Conclusion

TACE and MWA were feasible and safe locoregional treatment modalities in patients with unresectable pancreatic cancer, demonstrating low complication rates in routine clinical practice. The reported outcomes reflect real-world results achieved with these treatment approaches in a retrospective setting and should be interpreted with appropriate caution. Nevertheless, this long-term single-center experience adds meaningful data to the limited literature on interventional oncologic therapies for pancreatic cancer and supports further prospective investigation.

## Data Availability

The data presented in this article are available on reasonable request from the corresponding author due to potential legal or ethical restrictions.

## Abbreviations

FOLFIRINOX: 5-fluorouracil, oxaliplatin, irinotecan and leucovorin
INR: International normalized ratio
IRE: Irreversible electroporation
MWA: Microwave ablation
RFA: Radiofrequency ablation
SIR: Society of interventional radiology
SPSS: Statistical package for the social sciences
TACE: Transarterial chemoembolization
